# Restoration of the growth of *Escherichia coli* under K^+^-deficient conditions by Cs^+^ incorporation via the K^+^ transporter Kup

**DOI:** 10.1038/s41598-017-02024-4

**Published:** 2017-05-16

**Authors:** Souichiro Kato, Yoshiki Kanata, Wataru Kitagawa, Teruo Sone, Kozo Asano, Yoichi Kamagata

**Affiliations:** 1 Division of Applied Bioscience, Graduate School of Agriculture, Hokkaido University, Kita-9 Nishi-9, Kita-ku, Sapporo, Hokkaido 060-8589 Japan; 2 Bioproduction Research Institute, National Institute of Advanced Industrial Science and Technology (AIST), 2-17-2-1 Tsukisamu-Higashi, Toyohira-ku, Sapporo, Hokkaido 062-8517 Japan

## Abstract

Biological incorporation of cesium ions (Cs^+^) has recently attracted significant attention in terms of the possible applications for bioremediation of radiocesium and their significant roles in biogeochemical cycling. Although high concentrations of Cs^+^ exhibit cytotoxicity on microorganisms, there are a few reports on the promotive effects of Cs^+^ on microbial growth under K^+^-deficient conditions. However, whether this growth-promoting effect is a common phenomenon remains uncertain, and direct correlation between growth promotion and Cs^+^ uptake abilities has not been confirmed yet. Here, we validated the growth promotive effects of Cs^+^ uptake under K^+^-deficient conditions using an *Escherichia coli* strain with an inducible expression of the Kup K^+^ transporter that has nonspecific Cs^+^ transport activities (strain *kup*-IE). The strain *kup*-IE exhibited superior growth under the Cs^+^-supplemented and K^+^-deficient conditions compared to the wild type and the *kup* null strains. The intracellular Cs^+^ levels were significantly higher in strain *kup*-IE than in the other strains, and were well correlated with their growth yields. Furthermore, induction levels of the *kup* gene, intracellular Cs^+^ concentrations, and the growth stimulation by Cs^+^ also correlated positively. These results clearly demonstrated that Cs^+^ incorporation via Kup transporter restores growth defects of *E. coli* under K^+^-deficient conditions.

## Introduction

Cesium (Cs) is a Group I alkali metal and generally exists as a monovalent cation (Cs^+^) in natural environments. Cs^+^ has comparable physicochemical properties with other alkali metal cations, in particular with potassium ion (K^+^), owing to their similar ionic radii. The radioisotopes of cesium, especially ^134^Cs and ^137^Cs, have acquired enormous ecological importance due to concerns of radioactive pollution arising from nuclear weapon testing and from intentional and unintentional discharge from nuclear power plants^1, 2^.

Although Cs is regarded as a non-essential element, Cs^+^ can be assimilated by living organisms, including bacteria, fungi, and plants^1–10^. This feature has offered a potential application of such organisms for recovery of radiocesium from contaminated environments. Uptake of Cs^+^ is known to be nonspecifically mediated by K^+^ transport systems^1, 5^. For example, *Eschericia coli* has three K^+^ transport systems, namely, Kdp, TrkA, and Kup (formerly TrkD)^11^, amongst which only Kup exhibits nonspecific Cs^+^-transporting activities, although the affinity for Cs^+^ is much lower than for K^+^ (ref. 12).

The physiological function of Cs^+^ in living organisms remains uncertain. High concentration of Cs^+^, generally above the mM order, exhibits toxic effects on plants and microorganisms^5, 13–15^. These toxic effects have been explained by the induction of K^+^ starvation and the intracellular toxicity of Cs^+^ (ref. 5). High concentrations of extracellular Cs^+^ competitively inhibit activities of K^+^ transporters^12, 16^. Furthermore, elevation of intracellular Cs^+^ concentration owing to nonspecific Cs^+^ uptake can cause efflux of intracellular K^+^ to maintain intracellular cation balance and dysfunction in some essential biochemical functions, including K^+^-dependent enzymes and stabilization of internal structures (e.g., ribosomes)^5^.

By contrast, there are a few reports on the promotive effects of Cs^+^ on microbial growth. Jasper^17^ reported that supplementation of Cs^+^ relieved growth defects of *Rhodopseudomonas capsulatus* strain Z-1 in K^+^-deficient conditions. Similarly, Tomioka *et al*.^7^ reported that two *Rhodococcus* strains exhibited growth under K^+^-deficient conditions only when Cs^+^ was supplemented to the growth medium. The putative explanation for the growth promotive effects is that Cs^+^ substitutes for part of the essential function of K^+^. The growth promotion by Cs^+^ has been reported only for the two non-model bacterial lineages that inherently have high Cs^+^ uptake abilities. Hence, the generality of this effect in other microorganisms remains uncertain. Furthermore, direct correlation between growth promotion and Cs^+^ uptake abilities (i.e., intracellular Cs^+^ concentrations) has not been confirmed yet.

In this study, we validated the growth promotive effects of Cs^+^ incorporation under K^+^-deficient conditions using an *E. coli* strain JW5609 with an inducible expression of Kup K^+^ transporter that has nonspecific Cs^+^ transport activities. We investigated the Cs^+^/K^+^ incorporation and the growth in a K^+^-deficient medium supplemented with Cs^+^ to verify the direct correlation between Cs^+^ incorporation and the restoration of growth.

## Results and Discussion

### Growth of *E. coli* under K^+^-limited conditions

**Figure 1:**
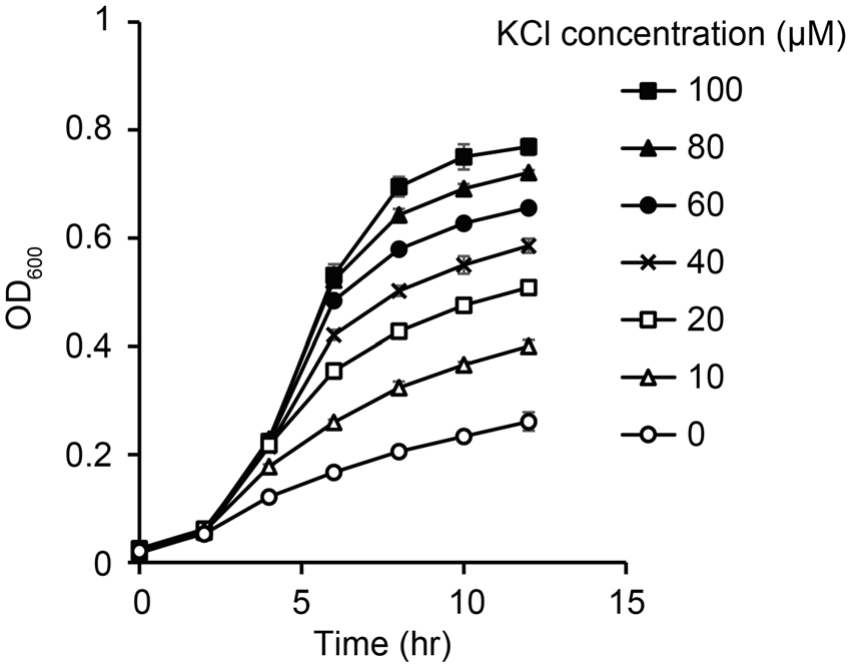
Growth of *E. coli* in the K^+^-limited conditions. *E. coli* WT was cultivated in the K^+^-free minimal medium supplemented with different concentrations of KCl and the OD_600_ values were monitored. Data are presented as means of three independent cultures, and error bars represent standard deviations.

Firstly, the growth properties of *E. coli* K12 wild type (hereafter referred to as WT) under K^+^-limited conditions were evaluated. The K^+^-free minimal medium was prepared by replacing K-containing reagents in the M9 minimal medium with Na-containing equivalents. The K^+^-free minimal medium contains a small amount of K^+^ (~8 μM) possibly derived from impurities. WT was cultivated in the K^+^-free medium supplemented with different concentrations of KCl (Fig. 1). The stimulation of growth was proportional to supplemented K^+^ from 0 to 100 μM, indicating that K^+^ was the growth-limiting factor under these conditions. Hereafter, the K^+^-free medium supplemented with 10 μM of KCl is defined as the “K^+^-deficient condition”, and the optical density at 600 nm (OD_600_) of the early stationary phases (after 12 hr cultivation) is defined as the “growth yield”.

### Construction of an *E. coli* strain with an inducible *kup* expression system

In order to investigate the effects of Kup expression levels on Cs^+^ incorporation, a transgenic *E. coli* strain with inductive Kup expression was constructed. The PCR-amplified *kup* gene was inserted downstream of the arabinose-inducible promoter P_BAD_ of the multicopy plasmid pBADGiiiA and transformed into the *E. coli* Δ*kup* strain. Hereafter, this strain is referred to as a strain *kup*-IE (Induced Expression). The *E. coli* Δ*kup* strain transformed with the pBADGiiiA empty vector was also constructed as a control, and is hereafter referred to as Δ*kup*-EV (Empty Vector).

**Figure 2:**
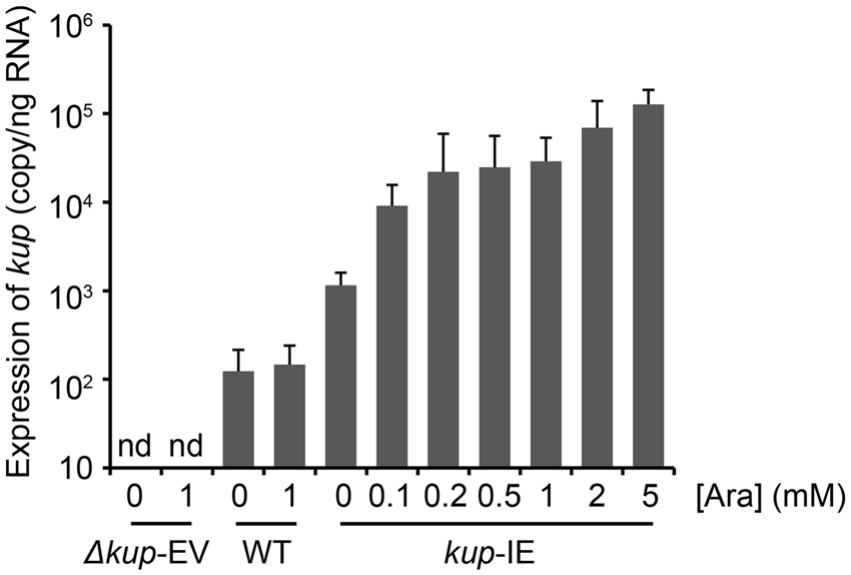
Arabinose-induced expression of *kup* in the *E. coli* strains. Each strain was cultivated in the K^+^-free minimal medium supplemented with 10 µM of KCl and different concentrations of arabinose. Total RNA was extracted from the early stationary phase cells and was subjected to the qRT-PCR analysis targeting the *kup* gene. Data are presented as means of triplicate experiments, and error bars represent standard deviations.

The *kup* expression levels in the WT, Δ*kup*-EV, and *kup*-IE strains with different arabinose concentrations were evaluated by quantitative RT-PCR (qRT-PCR) (Fig. 2). The *kup* expression levels in Δ*kup*-EV and WT were not significantly altered by the addition of arabinose, and were under the detection limit (<10 copies/ng RNA) and approx. 10^2^ copies/ng RNA, respectively. In the *kup*-IE strain, the *kup* expression level was significantly increased from approx. 10^3^ to 10^5^ copies/ng RNA by increasing the arabinose concentration from 0 to 5 mM. These results ascertained that arabinose-induced expression of *kup* was achieved and quantifiable in the *kup*-IE strain.

### The effects of Cs^+^ on growth under K^+^-deficient conditions

**Figure 3:**
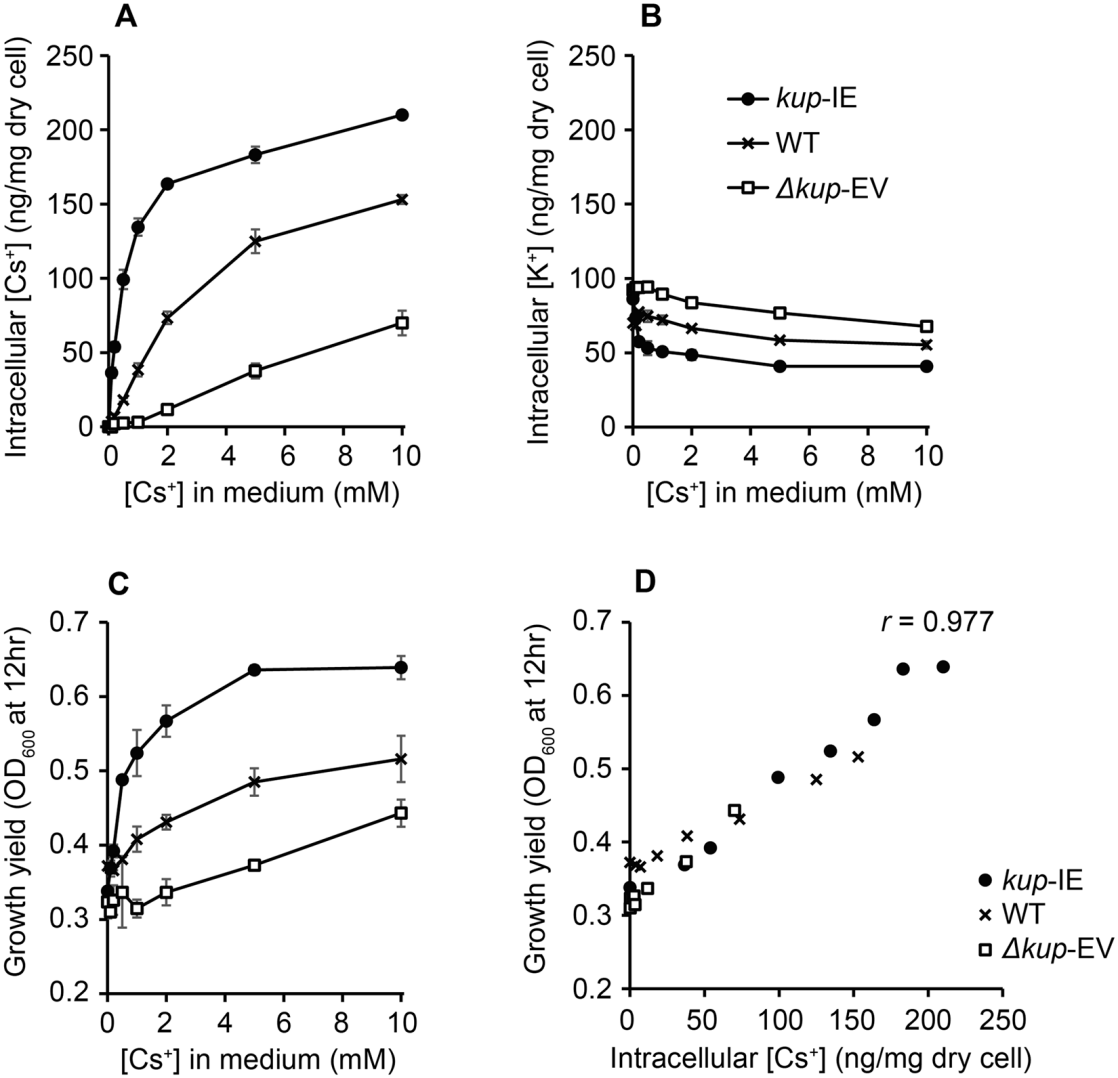
The effects of Cs^+^ on the growth of the *E. coli* strains under K^+^-deficient conditions. The intracellular Cs^+^ (**A**) and K^+^ (**B**) concentrations, and the growth yields (**C**) were measured after 12 hr cultivation in the K^+^-deficient medium supplemented with 1 mM of arabinose. (**D**) The growth yields were plotted against corresponding intracellular Cs^+^ concentrations. Data are presented as means of three independent cultures, and error bars represent standard deviations.

In order to investigate the effects of Cs^+^-uptake on growth under the K^+^-deficient conditions, WT, Δ*kup*-EV, and *kup*-IE were cultivated in the K^+^-deficient medium supplemented with 1 mM of arabinose and 0 to 10 mM of CsCl. The growth yields and intracellular [Cs^+^] and [K^+^] were determined after 12 hr of cultivation (Fig. 3). The intracellular [Cs^+^] levels of all strains elevated with increasing [Cs^+^] in the medium (Fig. 3A). It should be noted that Δ*kup*-EV also takes up Cs^+^ under high extracellular [Cs^+^] conditions, probably via nonspecific cation transport systems, as has been reported^12^. The intracellular [Cs^+^] levels under all CsCl supplemented conditions tested were *kup*-IE > WT > Δ*kup*-EV, suggesting that higher expression levels of Kup lead to more Cs^+^ uptake. By contrast, the trends of the intracellular [K^+^] were almost same among the three strains; the intracellular [K^+^] showed a small decrease with increasing [Cs^+^] in the medium (Fig. 3B). The intracellular [K^+^] levels under the CsCl supplemented conditions were inversely related to the Kup expression levels, *i.e*., Δ*kup*-EV exhibited the highest, followed by WT and *kup*-IE. Compared to the intracellular [Cs^+^], however, differences in the intracellular [K^+^] levels among the three strains were not so conspicuous. These results demonstrate that expression of Kup confers Cs^+^-uptake ability on *E. coli*, which is consistent with the previous report^12^.

Supplementation of CsCl stimulated the growth of all *E. coli* strains (Fig. 3C). The growth yields under the CsCl supplemented conditions were *kup*-IE > WT > Δ*kup*-EV, suggesting that the extent of growth promotion correlates with the *kup* expression levels (*i.e*., intracellular [Cs^+^] levels). Indeed, the growth yields and intracellular [Cs^+^] were well-correlated (*r* = 0.977, Fig. 3D). These results clearly demonstrate that Cs^+^ incorporation *via* the Kup transporter relieves growth inhibition under the K^+^-deficient conditions.

### The effects of Kup expression levels on the growth promoting effects of Cs^+^

**Figure 4:**
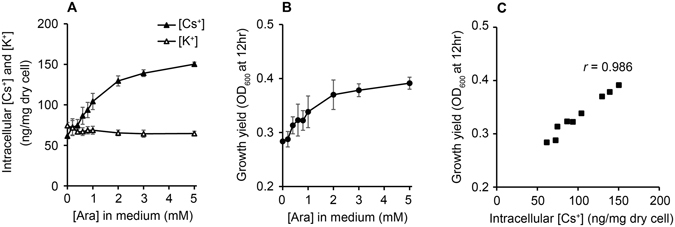
The effects of the induction of *kup* expression by arabinose supplementation on the growth stimulation of Cs^+^. The intracellular Cs^+^ and K^+^ concentrations (**A**) and the growth yields (**B**) were measured after 12 hr cultivation of the *E. coli kup*-IE strain in the K^+^-deficient medium supplemented with 1 mM of CsCl. (**C**) The growth yields were plotted against corresponding intracellular Cs^+^ concentrations. Data are presented as means of three independent cultures, and error bars represent standard deviations.

In order to further confirm the relationship between the expression levels of Kup and growth under the K^+^-deficient conditions, the *kup*-IE strain was cultivated in the K^+^-deficient medium supplemented with 1 mM of CsCl and 0 to 5 mM of arabinose (Fig. 4). The intracellular [Cs^+^] levels elevated by increasing the arabinose concentration in the medium, while the intracellular [K^+^] levels was not significantly affected by arabinose (Fig. 4A). The growth yields were also elevated by increasing the arabinose concentration (Fig. 4B). The growth yields and intracellular [Cs^+^] were well-correlated (*r* = 0.986, Fig. 4C), which confirms the assumption that growth under the K^+^-deficient conditions can be facilitated by Cs^+^ incorporation *via* Kup.

### Implication

Cytotoxic effects of Cs^+^ on physiological function have been well known^5, 13–15^. By contrast, this study demonstrated that Cs^+^ has growth-promoting effects on *E. coli*, at least under K^+^-deficient conditions. These contradicting phenomena can be accounted for by the analogy between K^+^ and Cs^+^. K^+^ has multiple essential physiological functions, including retention of pH and osmolality homeostasis, activation of some intracellular enzymes, and stabilization of internal structures (e.g., ribosomes)^5, 18^. Cs^+^ is predicted to substitute for part of K^+^ functions (probably retention of homeostasis), which causes restoration of growth under K^+^-deficient conditions. By contrast, Cs^+^ would work as a competitive antagonist for essential K^+^ functions (probably activation of K^+^-dependent enzymes), which accounts for the cytotoxicity. Further investigation is required to fully understand such duality in the physiological function of Cs^+^.

The stimulation of microbial growth by incorporation of Cs^+^ is expected to have crucial roles in the development of new biotechnologies for application in bioremediation of radiocesium. There have been some reports on the development of plant K^+^ transport systems with reduced affinity to Cs^+^ by molecular evolution engineering methods, with the aim of generating crop plants that accumulate less radiocesium^19, 20^. In these studies, mutated K^+^ transporters were expressed in yeast cells and screened for less Cs^+^ uptake activities using cytotoxicity of incorporated Cs^+^ as the indicator. By contrast, development of K^+^ transport systems with increased Cs^+^ transport, which would useful for bioremediation of radiocesium, has not been reported so far. The probable reason is that suitable methods for screening of organisms with high Cs^+^-uptake activities have not been available. Novel insights revealed in this study, *i.e*., acceleration of *E. coli* growth by incorporation of Cs^+^ under K^+^-deficient conditions, will be applicable to such screening. For instance, selection of *E. coli* strains expressing randomly mutated K^+^ transporters under K^+^-deficient and Cs^+^-supplemented conditions will allow us to acquire the Cs^+^-transporting variants.

### Conclusion

This study is the first to demonstrate that Cs^+^ incorporation restores growth defects of *E. coli* under K^+^-deficient conditions. While growth promoting effects of Cs^+^ have previously been reported^7, 17^, these studies utilized non-model microorganisms with relatively high Cs^+^-uptake abilities and assessed the effects of Cs^+^ by simply altering amounts of Cs^+^ supplemented to the K^+^-deficient media. On the other hand, we successfully observed a clear correlation between intracellular Cs^+^ concentrations and growth yields under K^+^-deficient conditions by constructing an *E. coli* strain that can be induced to expresses the Cs^+^-transport system (Kup). Further studies on the microbial incorporation of Cs^+^ and its growth promotive effects will shed light on the novel aspects of the physiological role of Cs and on the development of novel biotechnologies relating to problems of radiocesium contamination.

## Methods

### Bacterial strains and culture conditions

*E. coli* strain K12 wild type (ATCC 12435) was purchased from American Type Culture Collection. *E. coli* Δ*kup* strain (JW5609-KC) was purchased from KEIO collection of National BioResourse Project. All strains were routinely cultured at 37 °C in Luria-Bertani medium (pH 7) comprised of 5 g of yeast extract, 10 g of tryptone, and 10 g of NaCl per liter with appropriate antibiotics. The K^+^-free minimal medium (pH 7) was used for the experiments under the K^+^-deficient conditions. The K^+^-free minimal medium contained the following chemicals (per liter): 15.2 g of Na_2_HPO_4_·12H_2_O, 3.44 g of NaH_2_PO_4_·2H_2_O, 0.5 g of NaCl, 1 g of NH_4_Cl, 247 mg of MgSO_4_·7H_2_O, 14.7 mg of CaCl_2_·2H_2_O, 10 mg of thiamine·HCl, and 1.8 g of glucose. K^+^ and Cs^+^ were separately supplemented to the K^+^-free medium from stock solutions. Intracellular K^+^ and Cs^+^ concentrations were determined by the method described previously^15^. The K^+^ and Cs^+^ were quantified by an atomic absorption spectrophotometer Z-5310 (Hitachi Kyowa Engineering). All culture experiments were conducted in triplicate and the student’s *t*-test was used for the statistical analyses.

### Construction of transgenic *E. coli* strains

The *E. coli kup* gene was PCR amplified using primers kup-InF-pBAD-F (CAG GAG GAA TTA ACC ATG AGC ACT GAT AAT AAG CAA) and kup-InF-pBAD-R (GGA GAC CGT TTA AAC TCA GAT TTC GAC CTG AGT AC). The expression vector, pBADGiiiA (Invitrogen) was also PCR amplified using primers pBAD-InF-F (GTT TAA ACG GTC TCC AGC TT) and pBAD-InF-R (GGT TAA TTC CTC CTG TTA GC). These two fragments were then combined by In-Fusion cloning (Clontech) strategy. The resultant plasmid, pBADGiiiA-kup was introduced into *E. coli* ∆*kup* by conventional electroporation method to generate a strain with arabinose-inducible expression of *kup* (Kup-IE).

### Quantification of the *kup* expression

The specific primers targeting partial *E. coli* kup gene (kup-RT-F; ACC CGG AAG CGA TTA AGA AC, kup-RT-R; GAC AAT CAC AAT CAC GAC CG) were designed with Primer3 software. Total RNA was isolated from *E. coli* cells using the SV Total RNA Isolation System (Promega) and purified using an RNeasy Mini kit (Qiagen) with DNase treatment (RNase-free DNase set, Qiagen) as described in the manufacturers’ instructions. The purified RNA was spectroscopically quantified using a NanoDrop ND-1000 spectrophotometer (Thermo Fisher Scientific). RT reactions were performed using ThermoScript RT-PCR System for First-Strand cDNA Synthesis (Thermo Fisher Scientific) as described in the manufacturer’s instruction with the kup-RT-R primer. Quantitative real-time PCR analysis on the cDNA samples was performed using a LightCycler 96 real-time PCR system (Roche) and THUNDERBIRD SYBR qPCR Mix (Toyobo) as described previously^21^. After an initial denaturation at 95 °C for 1 min, targets were amplified by 40 cycles of denaturation for 10 sec at 95 °C, followed by annealing and extension for 4 min at 72 °C. Fluorescence was measured at the end of the extension step. The PCR amplicons were assessed by a melting-curve analysis to check for successful amplification. At least two separate trials were conducted for each cDNA sample. Standard curves were generated with serially diluted PCR products amplified using the same primer set.
